# Clinical manifestations in Egyptian Pompe disease patients: Molecular variability and enzyme replacement therapy (ERT) outcomes

**DOI:** 10.1186/s13052-025-01837-8

**Published:** 2025-01-23

**Authors:** Mohamed Abdelghafar Hussein, Heba ElTaher, Ranim Mahmoud, Donia Sobh, Mohammad Al-Haggar

**Affiliations:** 1 Pediatrics Department, faculty of medicine, Kafrelsheikh University, Kafrelsheikh, Egypt; 2 Pediatrics Department, Genetics Unit, Mansoura University, Mansoura, Egypt; 3 Radiodiagnosis Department, Mansoura University, Mansoura, Egypt

**Keywords:** Pompe disease, Genotypes, Cardiac magnetic resonance imaging (CMR), ERT

## Abstract

**Background:**

Pompe disease is a rare genetic disorder caused by a deficiency of the enzyme acid alpha-glucosidase. This condition leads to muscle weakness, respiratory problems, and heart abnormalities in affected individuals.

**Methods:**

The aim of the study is to share our experience through cross sectional study of patients with infantile-onset Pompe disease (IOPD) with different genetic variations, resulting in diverse clinical presentations. We evaluated their phenotype, genotype, radiological and laboratory findings including their cross-reactive immunologic material (CRIM) status. Infantile Pompe disease was diagnosed by measurement of the activity of the enzyme alpha-glucosidase. The diagnosis was confirmed by molecular genetic testing using PCR amplification and sequencing of the acid alpha-glucosidase (*GAA*) gene. Routine two-D echocardiography, and multi-parametric ECG-gated cardiac magnetic resonance imaging (CMR) were done to patients six months after starting enzyme replacement therapy (ERT).

**Results:**

The results of our study revealed different genetic mutations among our patients, different CRIM status and also CMR abnormalities. CMR imaging revealed abnormalities in all cases that underwent the procedure, including myocardial and vascular changes, with feature tracking indicating issues across all parameters and LGE suggesting fibrosis. The patient with a positive immune response had the most severe cardiac abnormalities, despite improvements in muscle weakness and motor skills from ERT. This underscores that delayed diagnosis and ERT can lead to irreversible heart damage from autophagy buildup.

**Conclusion:**

Pompe disease has various clinical presentations and results in significant CMR findings, which can be attributed to different genetic mutations. Early initiation of enzyme replacement therapy in infantile-onset Pompe disease is important to maximize its benefits.

**Supplementary Information:**

The online version contains supplementary material available at 10.1186/s13052-025-01837-8.

## Introduction

Pompe disease is an inherited, autosomal recessive lysosomal storage disorder caused by mutations in the gene coding for acid alpha-glucosidase (GAA). This consequently leads to the progressive lysosomal accumulation of glycogen in multiple tissues, especially cardiac and skeletal muscles. The global incidence of Pompe disease ranges from 1 in 14,000 to 1 in 300,000 live births which depends on the race and geographic region [1]. More than 450 mutations have been reported affecting the *GAA* gene on chromosome 17 q25. *GAA* gene is ∼28 kb long and contains 20 exons. Different ethnic groups have different common mutations, such as the c.-32-13T > G splicing mutation in Caucasian patients, p.R854X mutation in African Americans, and the p.D645E mutation in Chinese patients [2].

Pompe disease could present with variable clinical phenotypes ranging from the severely progressive infantile form which could be lethal to a slowly progressive late-onset form. Its clinical course is largely dependent on the type of genetic mutation and the resulting level of residual GAA activity., Hence, the disease can present at different ages with varying severity and system affection [3].The correlation between genotype and phenotype in Pompe disease (PD) is complex, with general trends observed in patient studies. Severe infantile PD is often linked to mutations that entirely abolish GAA protein expression or significantly reduce it, resulting in minimal residual activity. Mild mutations on one allele can mitigate the severity of the classic phenotype. However, establishing a direct link between specific mutations and clinical outcomes is challenging due to the prevalence of unique mutations and their occurrence in compound heterozygosity, limiting accurate predictions for individual cases [4–6].

Pompe disease is generally classified based on the age of onset and the presence or absence of cardiomyopathy into two main types: Classic infantile onset Pompe disease (IOPD) and late onset type (LOPD). IOPD is the most severe type with an age of onset at ≤ 12 months, progressive hypertrophic cardiomyopathy, left ventricular outflow obstruction, muscle weakness, hypotonia, respiratory distress, and progressive loss of independent ventilation. Breathing difficulties, feeding problems, and macroglossia are common manifestations. Affected patients usually have significant motor developmental delay in the form of inability to roll over, sit, or stand. Only a small percentage of untreated patients survive beyond 1 year of age; the main causes of death are cardiac and respiratory failure [7]. Less severe late-onset Pompe disease (LOPD) can manifest at any time after 12 months of age. Childhood, juvenile and adult-onset are examples for the late onset type. Patients with LOPD usually do not have significant cardiac involvement. They commonly present with slowly progressive proximal limb-girdle myopathy which leads to profound muscle weakness, wasting, wheelchair dependency, and respiratory failure due to the affection of the diaphragm [8]. Diagnosis of Pompe disease is based on the typical clinical manifestations, the determination of GAA enzyme activity deficiency in muscle or skin fibroblasts using dried blood spot (DBS) samples and confirmation by *GAA* mutation analysis [9].

Enzyme replacement therapy (ERT) with recombinant human acid alpha-glucosidase is administered to supplement or replace the defective enzyme and slow disease progression [10]. Clinical studies have shown significant improvement in cardiac muscle function more than skeletal muscles with ERT particularly in children. Thus, early identification of the affected individuals is important to initiate ERT as early as possible. The response to ERT depends on the age of treatment initiation, the degree of muscle affection and the original cross-reactive immunologic material (CRIM) status. CRIM-positive infants, who have residual GAA enzyme activity and immune tolerance to the GAA protein, generally respond well to ERT. However, CRIM-negative patients, who lack immune tolerance to GAA, typically have a poor response to ERT, although this response can be improved with immunomodulatory therapy.

In this cross-sectional study, we provide an analysis of eight patients with classic infantile-onset Pompe disease (IOPD) who have undergone ERT. This study aims to illustrate the variations in clinical presentation, laboratory, radiological findings, molecular variants, and CRIM status, as well as the impact of ERT on disease outcomes.

## Patients and methods

This was a single center cross sectional study that included eight patients with IOPD diagnosed and treated at the Mansoura University Children’s Hospital in the period from November 2021 and December 2022. Informed consent was obtained from the patients’ guardians. All experimental protocols were approved from the ethical committee in Mansoura university (IRB number MD.21.01.404).

### Inclusion criteria

#### Diagnosis

Patients must be diagnosed with infantile-onset Pompe disease (IOPD) by.

- Age: Patients with symptoms started before the age of 1 year [11].
- Clinical Symptoms: Patients must present with one or more of manifestations of Pompe disease: Generalized muscle weaknes, elevated creatine phosphokinase (CPK) level, hypotonia, hepatomegaly, hypertrophic cardiomyopathy, respiratory distress.
- Enzyme Activity: Patients must have confirmed quantitative blood alpha-glucosidase enzyme activity levels greater than 2 µmol/hr/l, measured by tandem mass spectrometry from dried blood spot [12].
- Molecular genetic diagnosis: It is confirmed by DNA extraction from dried blood spot, PCR amplification and sequencing of all coding exons and flanking inotropic regions of *GAA* gene and variants as described by the Human Genome Variation Society HGVS recommendations for the description of sequence variants [13].

#### Ethical compliance

Informed consent must be obtained from the patients’ guardians.

### Exclusion criteria

1. Alternative Diagnoses: Patients with conditions that may mimic Pompe disease or other myopathies not related to IOPD.
2. Incomplete Data: Patients with incomplete clinical, biochemical, or genetic data that precludes accurate diagnosis.
3. Age: Patients outside the age range typically associated with Pompe disease (e.g., older than 12 months at diagnosis).

CRIM status was evaluated by assessing the reactivity of monoclonal and/or polyclonal anti-rhGAA antibodies pool that can detect both native and recombinant GAA protein bands. This was performed using western blot analysis on fibroblast cells extracts. If none of the GAA proteins forms are identified, patients are classified as CRIM-negative [14].

### Cardiac imaging

#### Echocardiography

Routine two-D echocardiography, tissue Doppler echocardiography and speckle tracking echocardiography were done for all patients in addition to using the Philips Affiniti 50 machine and Q LAB software for imaging analysis.

Parameters of Echocardiography were validated against the reference values published by the American Society of Echocardiography [15].

#### Cardiac Magnetic Resonance Imaging (CMR)

Multiparametric ECG– gated Cardiac magnetic resonance imaging (CMR) was performed 6 months after ERT for all cases using 1.5 T Philips, (best, The Netherlands) using a commercially available cardiac software. Quantification of the ventricular functions and volumes was done on standard cine images and tissue characterization was done using T1 mapping and late gadolinium enhancement sequences. Myocardial deformation was assessed using tissue tracking CMR done on standard cine imaging using CVI42 software. Phase contrast CMR was useful to assess aortic flow and distensibility and by using the following equation of aortic distensibility (AD), AD = aortic maximum area-aortic minimum area)/(pulse pressure *aortic minimum area) [16].

CMR parameters were validated against the reference values published by the Journal of Cardiovascular Magnetic Resonance [17, 18].

### Statistical analysis

Statistical analysis was done using the SPSS program version 22. Descriptive statistics were used as appropriate. and interpreted using t-test for parametric values while Mann Whitney test was used for non-parametric independent samples and Wilcoxon rank for two-related samples and the results were explained as significant if (p value is ≤ 0.05).

## Results

### Clinical characteristics

Eight cases (five males and three females) were included in this cross sectional study with a mean age of 7.43 ± 2.7 months at diagnosis and ERT have been started once diagnosis is confirmed. Six cases were the products of consanguineous marriages. Two patients had a family history of IOPD. All cases had IOPD and presented with hypotonia, subsequent motor developmental delay, hepatomegaly, and cardiac decompensation (table: 1).

**Table 1:** Molecular variability and CRIM status of patients included in our study

Patient	Gender	CRIM	Molecular
1	Female	Positive	Homozygous missense mutation at codon 702 for Arginine c.[2104 C > T]; [2104 C > T] and (*p*.[Arg702Cys]; [Arg702Cys]).
2	Male	Negative	Homozygous missense mutation at codon 854 for Arginine [2560 C > T] (*p*.[Arg854Ter])
3	Male	Negative	(Homozygous missense mutation at codon 335 for Leucine c.[1064T > c]; [ 1064T > c] and (p.[Leu335Pro]; [Leu335Pro])
4	Female	Negative	(Homozygous frameshift mutation c.1464upc(p.(Asp489Argfs*17)).,
5	Male	Negative	Missense mutation in two variants p.Gly665, p.Gly665Arg)
6	Male	Negative	Homozygous missense mutation at codon 854 for Arginine [2560 C > T] (p.[Arg854Ter])
7	Female	negative	Homozygous non sense (stop) mutation in c.2560 C > T [p.(Arg854*)]
8	Male	positive	Compound heterozygous mutation in (c,-32-13T > G) and c,1856G > A [p.Ser619Asn)].

### Biochemical data at the time of presentation

#### Biochemical profile

LDH, SGPT, CPK were elevated in all cases (1308. IU/l ± 134.635, 333u/ml ± 120.30 and 720.25U/L ± 121.33 respectively).

#### Enzyme assay

Alpha glucosidase showed low activity in all cases at the time of presentation (0.175 µmol/L/h ± 0.119) (cut-off value > 2.0 µmol/L/h).

#### Mutational analysis

Various pathogenic variants were found in our patients where homozygous mutation at codon 854 for Arginine [2560 C > T] (p. [Arg854Ter]; was detected in three unrelated patients. Homozygous missense mutation at codon 702 for Arginine c.[2104 C > T]; [2104 C > T] and (p.[Arg702Cys]; [Arg702Cys]), missense mutation in two variants p.Gly665, p.Gly665Arg), homozygous frameshift mutation c.1464 (p.(Asp489Argfs*17), homozygous missense mutation at codon 335 for Leucine c.[1064T > c]; [1064T > c] and (p.[Leu335Pro]; [Leu335Pro]) and there was compound heterozygous mutation in one patient in the form of (c,-32-13T > G) and c,1856G > A(p.Ser619Asn).

**Fig. 1:**
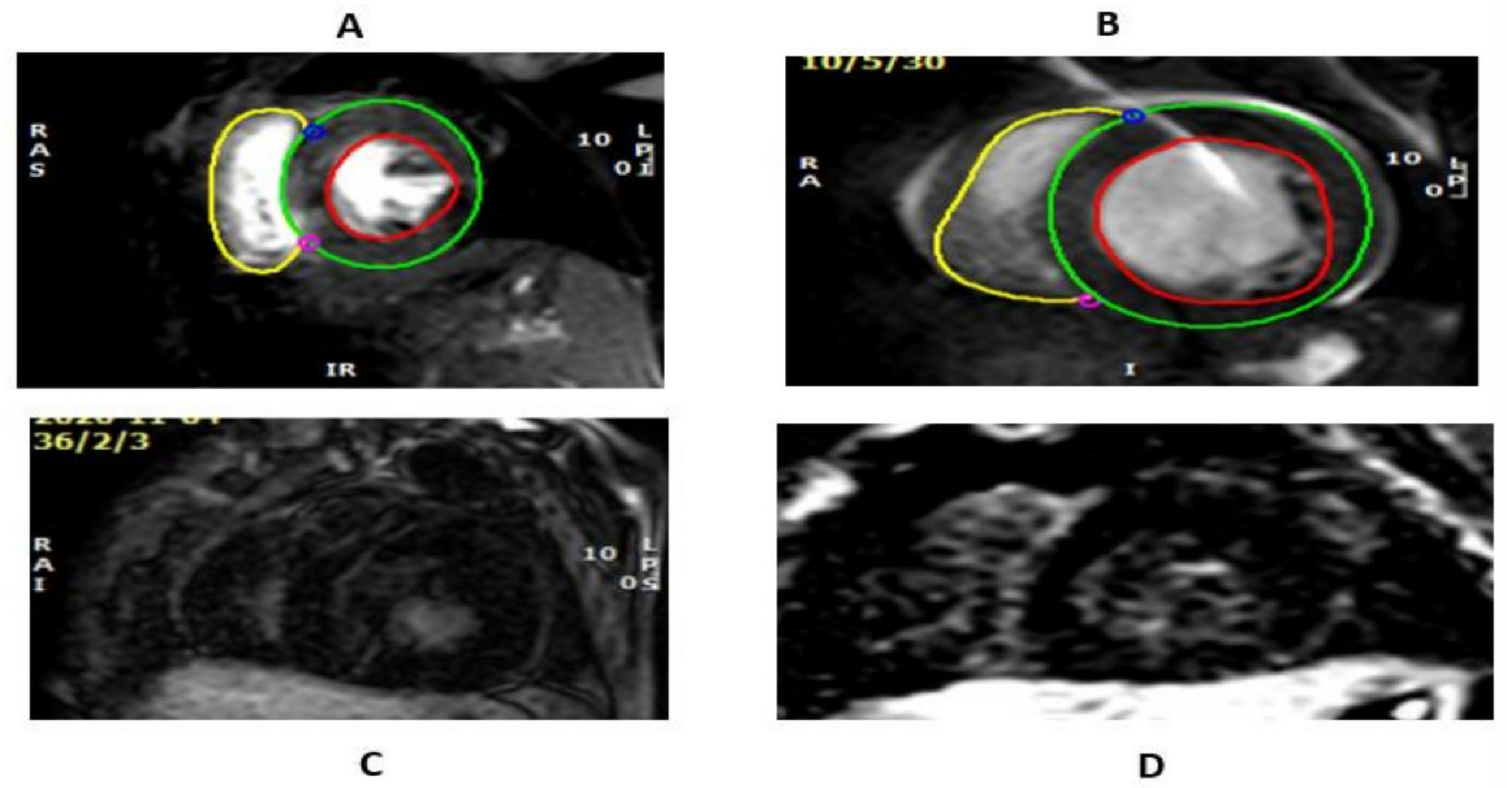
Case with homozygous p.[Arg702Cys] shows more hypertrophied left ventricle and more LGE (**A** and **C**) as compared to male patient with a male infant with homozygous mutation: c.[2560 C > T]; [2560 C > T] and (p.[Arg854Ter]; [Arg854Ter]) (**B** and **D**)

CRIM status was positive in two patients. The first one who had mild hypotonia and mild developmental delay, but his cardiac condition was more severe as regard to CMR (Fig. 1). The other one exhibited moderate hypotonia accompanied by severe cardiopulmonary impairment, which precluded her from undergoing CMR due to anesthetic considerations. Conversely, the other six patients who presented with severe hypotonia and global motor delay were CRIM negative.

#### Echocardiography

Tissue Doppler, speckle tracking echocardiography and two-D conventional echocardiography revealed impaired diastolic function and deformed myocardium as shown in (table: 2).

All patients had received ERT at a dose 20 mg/kg every two weeks. Unfortunately, all cases died at a mean age 17.0 months ± 2.07 by cardiac compromise and or respiratory failure 4-6 months after ERT despite of appropriate management with ERT, and immunomodulation for CRIM negative patients.

**Table 2:** Echocardiographic parameters in cases in relation to reference values

	LVEF	IVSd	IVSs	LVPW	E’	A^’^	E^’^/A^’^	LVGLS	LVGCS	LVGRS
Patients	47.3 ± 3.2	0.89 ± 0.097	1.0 ± 0.103	1.01 ± 0.16	8.2 ± 0.48	7.03 ± 0.28	1.16 ± 0.10	12.8 ± 1.96	11.6 ± 1.4	19.6 ± 2.9
Reference values	55 ± 5.5	0.28–0.57	0.40–0.70	0.49–0.81	11.9 ± 1.2	6.8 ± 0.7	1.6 ± 0.57	21.5 ± 1.2	20.2 ± 1.5	33.1 ± 2.5
P value	0.000	0.000	0.000	0.000	0.000	0.160	0.000	0.000	0.000	0.000

#### CMR

**Fig. 2:**
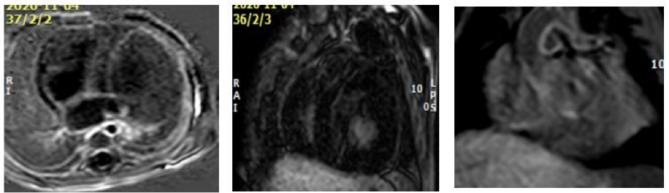
**A**: Phase sensitive inversion recovery image in female infant with Pompe disease shows septal late gadolinium enhancement in apical part of left ventricle denoting non ischemic myocardial injury (yellow arrow), **B**: Late gadolinium enhancement in anteroseptal in apical part of left ventricle myocardium in the same infant (blue arrow), **C**: Aortic wall enhancement in the same infant in inversion recovery sequence (red arrow)

**Fig. 3:**
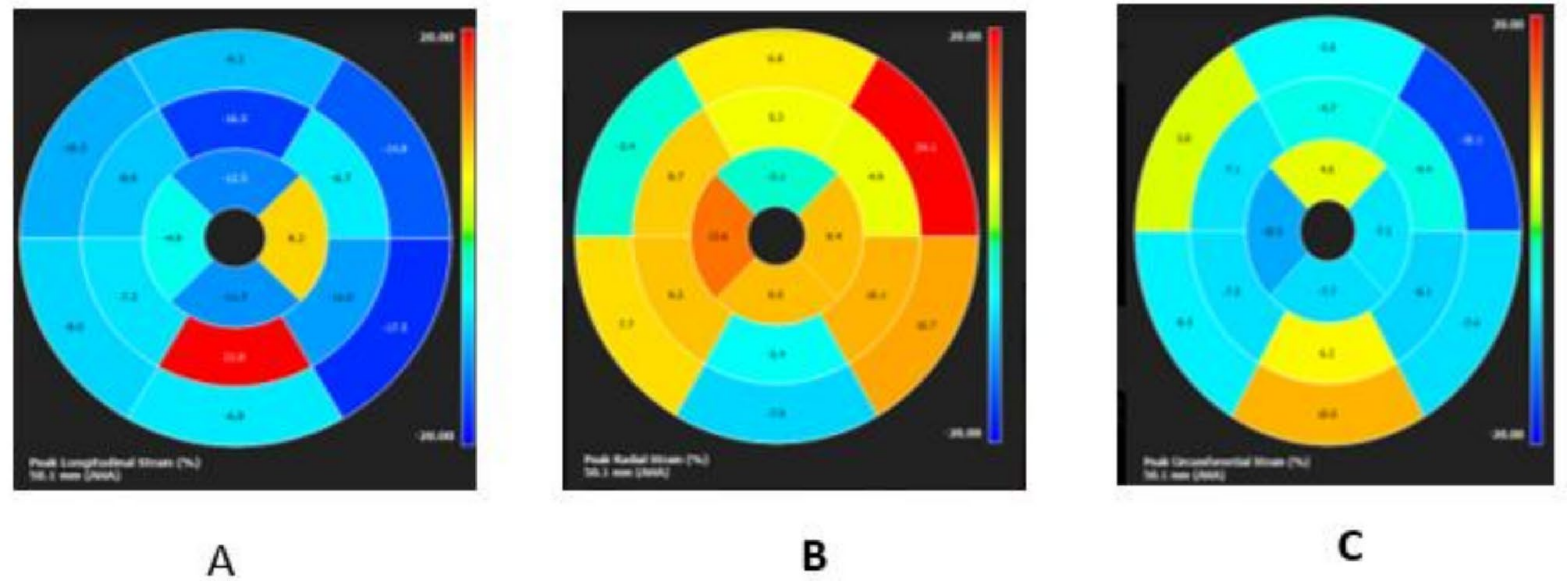
Feature tracking CMR of the same infant shows affection in all strains denoting significant myocardial deformation (**A**: longitudinal strain, **B**: Radial strain, **C**: circumferential strain)

CMR revealed affection of all parameters of feature tracking CMR and increase in global T2 time, with increased extracellular volume (ECV). In addition, all cases showed late gadolinium enhancement (LGE) with subsequent myocardial fibrosis (Table 3& Figs. 1-3).

**Table 3:** CMR parameters in cases in relation to reference values

	LVEF	LVMI	LVEDVI	LVESVI	LVGLS	LVGRS	LVGCS	ECV	LGE	AD (10^− 3^)	Global T2 time
patients	40.4 ± 5.9	116.2 ± 14.02	118.02 ± 41.2	74.8 ± 30.5	11.4 ± 2.7	12.6 ± 4.02	11.24 ± 3.11	33.5 ± 15.3	8.07 ± 6.07	4.3 ± 2.01	65 ± 4.3
Ref values ( at z scores − 2 - +2) at mean BSA	66 ± 9.2	65.2 ± 1.9	65.8 ± 0.55	27.2 ± 1.35	21.4 ± 0.34	22.8 ± 0.94	23.4 ± 0.83	28.2 ± 0.8	0.7 ± 0.07	9.3 ± 0.5	56.5 ± 1.61
P value	0.008	0.000	0.000	0.000	0.000	0.000	0.000	0.42	0.000	0.001	0.000

Regarding vascular assay. All cases showed decreased aortic dispensability with two cases having considerable LGE in aortic root wall (Table 3; figure 2).

The CRIM-positive patient that had CMR showed marked deterioration in all parameters compared to CRIM-negative ones (Table 4).

**Table 4:** CRM results in CRIM positive and negative patients

	CRIM positive patients	CRIM negative patients	*P* value
LVEF	28.9	36.1 ± 14	0.11
LVMI	145	111.8 ± 6.5	0.005
LVEDVI	179.2	107.4 ± 34.04	0.11
LVESVI	127.4	66.6 ± 23.5	0.48
LVGLS	6.5	12.2 ± 1.8	0.038
LVGRS	4.5	14 ± 1.9	0.007
LVGCS	5.11	12.2 ± 1.69	0.011
ECV	40.9	32.7 ± 16.4	0.645
LGE	20.0	6.3 ± 3.3	0.012
AD(10^− 3^)	1.3	4.8 ± 1.6	0.12
Global T2 Time(MS)	74.0	63.0 ± 1.9	0.003

## Discussion

Diagnosis of IOPD in Egypt is initially based on clinical index of suspicion as national newborn screening for Pompe disease has not been applied in Egypt yet. Hypotonia with muscle weakness, respiratory insufficiency with repeated respiratory infections, feeding difficulties, failure to thrive and cardiac problems are non-specific clinical features which may lead to delayed diagnosis. Idiopathic hypertrophic cardiomyopathy, spinal-muscular atrophy, neurogenetic and other metabolic disorders could have similar clinical presentations [19].

To date, over 550 distinct *GAA* mutations have been detected; about 450 of them are thought to be pathogenic [20].We reported eight Egyptian infants of different descendants with IOPD with different molecular diagnosis and distinct clinical manifestations of variable severity.

In IOPD, it has been shown that CRIM-negative status is associated with reduced overall survival and poor outcome [14]. In our study the only two CRIM positive cases presented with mild to moderate hypotonia and motor delay, they showed improvement of gross motor developmental milestones with ERT and physiotherapy but unfortunately their cardiac status were severely affected. The remaining CRIM negative cases were presented with severe hypotonia and marked global motor delay with no satisfactory improvement of gross motor skills inspite of appropriate treatment with ERT and Immunomodulation.

Immunomodulation was shown to induce tolerance in patients with CRIM-negative status especially in the naïve setting. We used the protocol of combination of rituximab with methotrexate ± intravenous gammaglobulins in the six CRIM negative patients.

Prognosis for IOPD patients depends on several factors, including not only *GAA* genotypes (and any resulting residual functional GAA activity) but also cross-reactive immunologic material (CRIM) status, CRIM-negative patients harbor *GAA* variants (nonsense, insertion, deletion, or indel) preventing native GAA protein synthesis or prematurely stop the protein product. In contrast, CRIM-positive patients produce some immunologically recognizable GAA protein. CRIM status can usually be predicted from genotypes [21].

In IOPD, accumulation of glycogen results in a continuous damage within the myocytes. In early stages the cells contain small, glycogen-filled lysosomes, but with disease progresses, glycogen continues to accumulate, these lysosomes increase in number and size, glycogen leaks into the cytoplasm, and mitochondrial structure becomes abnormal. Eventually, cytoplasmic glycogen predominates, and there is a complete loss of fibril and sarcoplasmic structure [22]. At this stage, muscle damage is irreversible, and the cells are incompatible with an effective response to ERT. The benefits of ERT are therefore greatest when initiated early in the disease course [23].

Based on Modified lake Louise criteria, CMR is now the gold standard for detection of non-ischemic myocardial inflammation and scarring in patient with inflammatory heart diseases including storage diseases as IPD [24, 25], presence of such myocardial LGE and aortic wall gadolinium enhancement in association with severe depressed left ventricular functions and more aortic stiffness denoting more cardiac and aortic root affection in patient with CRIM positive status. This was enforced by more affected tissue tracking CMR results which could detect early myocardial deformations.

For better cardiac description, echocardiography has the advantage of easy availability, high temporal resolution and no need for long time anesthesia, however interference from lung and bone prevent reasonable description of blood pool and endocardium interface [26]. On the other hand, CMR has the advantage of precise definition of myocardium from blood pool and wide acoustic window [27], but due to effect of respiratory motion on CMR images and subsequent lower signal to noise ratio, sedation and prolonged anesthesia is mandated [28, 29].

Anesthesia in poorly controlled cases of Pompe disease may result in myocardial ischemia and arrhythmia [28], so we postponed doing CMR to 4–6 months after ERT and after assurance of anesthetic fitness under supervision of pediatric cardiac anesthesia.

Many studies compared left ventricular measurements from CMR with cardiac weights from autopsy and concluded that CMR has the power of precise description of left ventricular measurement especially in cases with left ventricular hypertrophy as Pompe disease [30, 31].

ECG gated SSFP sequence was found to be helpful in yielding adequate imaging for calculating LV mass with limited respiratory artifact,thus helpful with less cooperative children and reduce time for anesthesia [28, 32].

CMR has also the ability to quantify myocardial fibrosis by late gadolinium enhancement, as it is known, Gadolinium is extracellular molecule which poorly accumulate in normal heart with low extracellular space and adjacent myocytes, but in cases with myocardial injury and subsequent fibrosis as in cases of Pompe disease, there would be accumulation of Gadolinium and these areas would be enhanced, quantification of these areas would make idea about the extent of fibrosis and myocardial viability [33, 34].

On the same line, increase extracellular space would affect inversion recovery time detected using T1 sequence, T1 time would be increased in cases with increase extracellular volume as in cases of pompe disease [35–39] and this explains the increase of extracellular volume in our cases.

Also, Accumulation of glycolipids in myocytes would prolong transverse relaxation of [H] ions and subsequent increase global T2 time [40] and this why T2 time was prolonged in our cases.

Although numerous studies have documented a favorable cardiac response to ERT, including reductions in ventricular hypertrophy and heart failure [41–43], they also noted the persistence of certain cardiac sequelae, such as arrhythmias, even following ERT [42, 44]. Furthermore, these studies emphasized the importance of early intervention with ERT, which appears to confer greater benefits, while late initiation of therapy may yield diminished advantages after accumulation of autophagy-related issues [43, 45–47].

Due to autophagic buildup, myocardial and skeletal fibrosis may be irreversible in some cases especially if treatment is delayed [47, 48], and this would explain why our cases showed this pattern of fibrosis and myocardial injury even after ERT.

CRIM status influences ERT response, with CRIM-negative patients lacking detectable glucosidase activity and having higher antibody titre, this suggests that CRIM-positive patients typically respond better [49]. However, in our study, CMR of CRIM positive case showed marked affection, despite improvements in hypotonia and motor milestones. Late diagnosis and delayed ERT likely lead to autophagic buildup and irreversible cardiac damage, ultimately resulting in death from cardiac decompensation [48].

To our knowledge, it is the first study to include molecular assay in Pompe disease and cardiac status by CMR that would affect ERT in African people.

## Conclusion and future therapeutic consideration

Pompe disease is a lysosomal storage disorder caused by a deficiency of the enzyme acid alpha-glucosidase. We demonstrated different clinical presentation and CMR findings which could be explained by different types of genetic mutation and different timing of initiation of ERT. In IOPD, accumulation of glycogen results in continuous damage within the myocytes so benefits of ERT are greatest when initiated early in the course of the disease.

## Electronic supplementary material

Below is the link to the electronic supplementary material.

Supplementary Material 1

## Data Availability

The datasets used and/or analyzed during the current study are available from the corresponding author on reasonable request.
