# Supplementary material for: Clinical manifestations in Egyptian Pompe disease patients: Molecular variability and enzyme replacement therapy (ERT) outcomes
Source: Ital J Pediatr. 2025 Jan 23;51:13. doi: 10.1186/s13052-025-01837-8 (PMC11756172; doi:10.1186/s13052-025-01837-8)
Supplement: Supplementary file 1 — Supplementary Material 1 [file 13052_2025_1837_MOESM1_ESM.docx]

Dear Dr. Angelo Selicorni

Thank you for the opportunity to revise our mansuscript entitled” 'Pompe Disease: From Pathophysiology to Management' (ITJP-D-24-00144)”. We sincerely appreciate the valuable feedback and suggestions from the reviewers, which have significantly improved the clarity and quality of our work. We have addressed all the points raised and made the necessary revisions to our manuscript. Below, we provide a point-by-point response to each comment.

Reviewer reports:

Reviewer #1: Heart involvement as well as the usefulness of heart NMR have been widely described The authors describe seven patients with infantile-onset Pompe disease (IOPD). Thesepatients had different genetic variations, resulting in diverse clinical presentations.

All patients underwent routine 2D echocardiography, and six months after initiating enzyme replacement therapy (ERT), they underwent multi-parametric ECG-gated cardiac magnetic resonance imaging (CMR) to assess the heart.

The authors underline the presence of a genotype phenotype correlation

Interestingly, the patient who tested positive for the specific immune response had the most severe cardiac abnormalities on CMR, despite showing improvement in muscle weakness and motor skills with ERT. This suggests that delayed diagnosis andinitiation of ERT can lead to irreversible heart damage due to autophagy buildup.

The authors conclude that in infantile onset Pompe disease, the continuous damage caused by glycogen accumulation in the cells underscores the importance of early initiation of enzyme replacement therapy to maximize its benefits

The results and the conclusion of the current study have already been widely reported.

However they confirm the importance of heart involvement and suggest the role of heart NMR in these patients.

The manuscrip worth publication

Minor changes

Reply: Thank you for your valuable and positive feedback; it has been truly encouraging.

Reviewer #2: Authors report their experience with seven patients with classic IOPD who have received ERT aiming to illustrate the variations in clinical presentation including the laboratory and radiological findings as correlated to the CRIM status , molecular variant, and the effect of ERT on the outcome of disease The authors aim to make correlations between molecular variants, cardiac status by CMR and ERT but there are no clear evidences reported to demonstrate the hypothesis

Reply:

Reply: thank you for your valuable feedback that would enhance our manuscript, your suggestions are very appreciated, regarding this point, many studies reported different observations regarding genotype-phenotype correlations, and we have included this in details with bibliography in 2^nd^ paragraph in introduction. Effect of CRIM Status on phenotypes were also reported in review of literature as included in 3^rd^ paragraph. Effect of ERT on outcome have been described in details in both introduction and discussion as reported in review of literature. And this supports our hypothesis to share our experience through cross sectional study of patients with infantile-onset Pompe disease (IOPD) with different genetic variations, resulting in diverse clinical presentations. We evaluated their phenotype, genotype, radiological and laboratory findings including and their specific immune response status (known as CRIM).

Language is poor and needs extensive corrections/rewriting The paper should be rewritten with major revisions,

Reply: we respect your feedback, language and paper have been revised by expert and rewritten in proper language.

some suggestions are reported below:

the title does not fit with the data reported in the text

Clinical manifestations in Egyptians Pompe disease patients: molecular variability and Enzyme replacement therapy outcomes.

the abstract is too long

Reply: we have compressed the abstract to focus more on the entire work

introduction: add what it is known in literature about the correlation genotype-phenotype in Pompe

Reply: appreciated!, done

disease Patients and Methods:

the same concepts are repeated twice, please shorten Line 17: correct the unit of measurement of enzymatic activity and add bibliographic reference of the enzyme activity method, add method of determination of CRIM status and the reference

Reply:

We have corrected all duplications as found in molecular, Biochemical, Echo, etc..

Unit of measurement of enzymatic activity has been included in addition to reference

Method of determination of CRIM status have been addressed with reference

Results: the age of starting ERT is not specified

Reply: Thanks for important notifications, ERT have been started once diagnosis, Age of diagnosis have been already included” with a mean age of 7.4 ± 2.7 at diagnosis”. We have corrected the statement to include time of start of ERT.

, in the mutations section it is not specified whether the genetic variants have already been reported in literature and therefore the bibliographical references

Reply: Genetic variations reported worldwide have been already mentioned in section of introduction as this parapgraph” More than 450 mutations have been reported in the GAA gene on chromosome 17 q25. GAA gene is ∼28 kb long and contains 20 exons. Different ethnic groups have different common mutations, such as the c.-32-13T>G splicing mutation in Caucasian patients, p.R854X mutation in African Americans, and the p.D645E mutation in Chinese patients “with reference (2)” but in the section of mutational analysis we reported genetic variations of our patients.

line 20: genetic variations ..which correlated with different clinical phenotypes, what do you mean?cardiac phenoptype ?

Reply:

Thank you for your important observation. This statement is indeed included in the results section of the abstract; however, we believe it does not accurately reflect our work, as we did not compare different clinical phenotype patterns across various genetic variations. Therefore, we have removed this statement. Instead, we focused on comparing cardiac presentations across different CRIM status patterns.

explain table 1: correct "neagtive" , "negativge"

Reply: Done

Discussion: the part about CRIM status is already known and is repeated too many times,

Reply :

Thank you for your important and valuable notice. We have revised this section and condensed the paragraphs in the discussion to eliminate redundancy

try to describe more thoroughly the different cardiac response to ERT

Reply:

We concur that this is an important point to address in the discussion section. We have included a paragraph that describes the cardiac response to ERT

“Although numerous studies have documented a favorable cardiac response to enzyme replacement therapy (ERT), including reductions in ventricular hypertrophy and heart failure, they also noted the persistence of certain cardiac sequelae, such as arrhythmias, even following ERT. Furthermore, these studies emphasized the importance of early intervention with ERT, which appears to confer greater benefits, while late initiation of therapy may yield diminished advantages after accumulation of autophagy-related issues”

**Notice:** Following a thorough self-review of our manuscript, all authors have agreed to include an eighth case characterized by a distinct molecular assay and CRIM positive status, which may have implications for outcomes within the same study period. This case was not initially included in the original manuscript due to the absence of Cardiac Magnetic Resonance (CMR) imaging, stemming from anesthetic considerations. However, we believe that, given the rarity of the disease, incorporating this case will enhance the study, despite the lack of CMR data, as other relevant parameters have been adequately addressed.
